# Long noncoding RNA DNAJC3‐AS1 promotes osteosarcoma progression via its sense‐cognate gene DNAJC3

**DOI:** 10.1002/cam4.1955

**Published:** 2019-01-16

**Authors:** Ridong Liang, Zezheng Liu, Zhixu Chen, Yang Yang, Yuejun Li, Zhifei Cui, Ajuan Chen, Zhenxue Long, Jinbin Chen, Jiachun Lu, Bin Huang, Qingchu Li

**Affiliations:** ^1^ Department of Orthopedics The Third Affiliated Hospital Academy of Orthopedics Southern Medical University Guangzhou China; ^2^ Department of Orthopedics The People's Hospital of Baise Baise China; ^3^ The First Affiliated Hospital The School of Public Health The Institute for Chemical Carcinogenesis Guangzhou Medical University Guangzhou China

**Keywords:** cisplatin, DNAJC3‐AS1, eIF2α, metastasis, osteosarcoma, proliferation

## Abstract

Long noncoding RNAs have been proved to play essential roles in tumor development and progression. In this study, we focused on DNAJC3‐AS1 and investigated its biological function and clinical significance in osteosarcoma. We detected the expression of DNAJC3‐AS1 in 30 pairs of matched osteosarcoma and adjacent nontumorous specimens and osteosarcoma cell lines and analyzed association between DNAJC3‐AS1 levels and clinicopathological factors. We found that DNAJC3‐AS1 expression was up‐regulated in osteosarcoma. High level of DNAJC3‐AS1 was correlated with high differentiated degree (*P* = 0.018) and advanced Enneking stage (*P* = 0.016). Mechanistically, DNAJC3‐AS1 enhanced cell proliferation, migration, and invasion in vitro and in vivo and reduced sensitivity of osteosarcoma to cisplatin. These effects of DNAJC3‐AS1 were reversed by down‐regulation of its sense‐cognate gene DNAJC3. Thus, DNAJC3‐AS1 promotes osteosarcoma development and progression by regulating DNAJC3 and might be a biomarker and therapeutic target for osteosarcoma.

## 1 INTRODUCTION

Osteosarcoma (OS) is the most common primary solid bone malignancy threatening health of children and adolescence.^1^, ^2^, ^3^ On account of lacking effective diagnostic methods in clinic, OS patients present high rate (approximately 20%) of lung metastases at first diagnosis.^4^ In the past 25 years, owing to combination therapy of surgical therapy and chemotherapy, the five‐year tumor‐free survival rate of OS has been raised up to 60%‐75%.^5^, ^6^ However, those who with metastasis and local recurrence still have a poor clinical prognosis.^7^, ^8^ Thus, it is exigent for clinical surgeons to find out effective strategies for early diagnosis, treatment, and prognosis of OS.

Results from high‐throughput transcriptome analysis have shown that 98% of the human genome can be transcribed into noncoding RNA. Among these noncoding RNAs, the long noncoding RNA (lncRNAs) with >200 nucleotides could not be translated into proteins.^9^, ^10^, ^11^ This does not, however, mean that lncRNAs are useless. Increasing evidence supports that lncRNAs involve in varieties of life activities, including embryonic development, cell growth, cell differentiation and apoptosis, and tumorigenesis, by regulating gene expression at the posttranscriptional and transcriptional levels.^12^, ^13^, ^14^, ^15^, ^16^, ^17^ In particular, lncRNAs act as oncogenes or tumor suppressors by epigenetic silencing, mRNA splicing, lncRNA‐miRNA interaction, lncRNA‐protein interaction, and lncRNA‐mRNA interaction. Yashar S. Niknafs et al demonstrated that DSCAM‐AS1 mediates tumor progression and tamoxifen resistance and identified hnRNPL as an interacting protein involved in the mechanism of DSCAM‐AS1 action.^18^ Chen et al reported that LncSox4 is required for liver TIC self‐renewal and tumor initiation. LncSox4 interacts with and recruits Stat3 to the Sox4 promoter to initiate the expression of Sox4, which is highly expressed in liver.^19^

In the present study, we discovered lncRNA‐DNAJC3‐AS1 expressed much more higher over tumor than normal tissue by bioinformatic analysis. DNAJC3‐AS1 locates at chromosome 13q32.1 with 2 exon counts. DNAJC3, also known as P58, HP58, and P58IPK, is the sense‐cognate gene of DNAJC3‐AS1. DNAJC3 belongs to heat shock protein family, which is a kind of conservative molecules that expresses extensively in cells of human and animals and protects cells against all kinds of damage.^20^ Michael A. Moses et al discovered the feasibility and potential benefit of targeting the Hsp40/Hsp70 chaperone axis to treat prostate cancer which is resistant to standard anti‐androgen therapy.^21^ DNAJC3 could also inhibit cell apoptosis by defending cells against endoplasmic reticulum (ER) stress via reduce the phosphorylation of eIF2α, which directly affects cellular biological response, such as cell adaptive damage or apoptosis.^22^, ^23^, ^24^, ^25^ Danmei Gao et al demonstrated that up‐regulation of p58IPK in ERp29 over‐expressing cells has a critical role in attenuating eIF2α phosphorylation and inhibiting the ATF4/CHOP/caspase‐3 pro‐apoptotic pathway, leading to enhanced breast cancer cell survival.^26^ Sophie J. Gilbert et al demonstrated that knockout of P58^IPK^, the cellular inhibitor of PKR and PERK, alters bone size and volume and leads to a degenerative joint phenotype.^27^ These studies revealed essential role of DNAJC3 in cell biology, while the biological function of DNAJC3‐AS1 in cells remains unclear. Moreover, the noncoding nature of DNAJC3‐AS1 was confirmed by coding‐potential analysis.^28^

In this study, we demonstrated that DNAJC3‐AS1 was up‐regulated in OS specimen and cell lines. Besides, we investigated the positive correlation between DNAJC3‐AS1 expression and clinicopathologic characteristics. What is more, we revealed that DNAJC3‐AS1 acted as a boost in the regulation of genesis and development of OS in vitro and *in mice*.

## 2 MATERIALS AND METHODS

### 2.1 Patient samples

Thirty pairs of matched fresh OS specimens and adjacent nontumorous specimens were collected from the Third Affiliated Hospital of Southern Medical University between 2015 and 2017. Specimens were snap‐frozen in liquid nitrogen immediately until RNA isolation. Diagnosis of OS has been confirmed pathologically during operation. In addition, complete clinical information and characteristics data of the patients were collected. This study was performed with the approval from the Ethics Committee of the Southern Medical University, and all patients had signed the written informed consent.

### 2.2 Cell culture

The human OS cell lines (HOS Cl#5[R‐1059‐D], Saos‐2) and osteoblast cell line hFOB1.19 were purchased from the Cellcook Biotech Company (Guangzhou, China), and HOS and SAOS‐2 were cultured in minimum essential medium (MEM) (Gibco, Life Technologies, Carlsbad, CA, USA) supplemented with 10% fetal bovine serum (FBS, Gibco, Life Technologies, Carlsbad, CA, USA), penicillin(100 U/mL), and streptomycin(100 μg/mL) in SANYO AUTOMATIC CO_2_ INCUBATOR (SANYO Electric Co., Ltd., Japan) at 37°C. hFOB1.19 was cultured in Dulbecco's modified Eagle's medium (DMEM) (Gibco, Life Technologies) supplemented with 10% fetal bovine serum (FBS, USA), L‐glutamine(150 mg/L), G418(0.3 mg/mL), penicillin(100 U/mL), and streptomycin(100 μg/mL) in SANYO AUTOMATIC CO_2_ INCUBATOR (SANYO Electric Co., Ltd., Osaka, Japan) at 33.5°C.

### 2.3 RNA extraction and real‐time PCR

Total RNA of cells or specimens was extracted by Trizol reagent (Invitrogen, CA), and then, the total RNA was reverse transcribed into cDNAs using SuperScript III First‐Strand Synthesis System (Invitrogen, Carlsbad, CA). And then, real‐time PCR reactions were performed by using SYBR PrimeScript RT‐PCR kit (Takara, Takara Biomedical Technology (Beijing) Co., Ltd., Beijing, China) with ABI PRISM 7900 HT system, the reaction was performed as follows: 95°C for 10 minutes; 40 cycles at 95°C for 15 seconds; and 60°C for 1 minute. Each assay above was performed in triplicate, and β‐actin was employed as endogenous control gen. The primer sequences used were as follows: DNAJC3‐AS1 forward: 5′‐AGCGATTGTGGAAGACCCTG‐3′; reverse: 5′‐ATTTCCCCTGGTAAGCGCAA‐3′; DNAJC3 forward: 5′‐GCCACACACCTTTCCTCCTC‐3′, reverse: 5′‐GCAGATCCACCAGGACTAGC‐3′; β‐actin forward: 5′‐GGCGGCACCACCATGTACCCT‐3′, reverse: 5′‐AGGGGCCGGACTCGTCATACT‐3′; U6 forward: 5′‐CTCGCTTCGGCAGCACA‐3′, reverse: 5′‐AACGCTTCACGAATTTGCGT‐3′; GAPDH forward: 5′‐GGTGAAGGTCGGAGTCAACG‐3′, reverse: 5′‐CAAAGTTGTCATGGATGHACC‐3′. The relative levels of gene expression were represented as ΔCt = Ct_gene _− Ct_reference_, and fold change of gene expression was calculated by the 2^−ΔΔCt^ method.

### 2.4 Subcellular fraction analysis

We extracted nuclear and cytoplasmic RNA by using the nuclear/cytoplasmic isolation kit (Biovision, San Francisco, CA). These RNAs were prepared for QRT‐PCR to determine the cellular localization of DNAJC3‐AS1.

### 2.5 Plasmid construction and transduction

The full‐length human DNAJC3‐AS1 cDNA and small hairpin RNA (sh‐RNA) are both synthesized by iGeneBio (Guangzhou, China), after synthesized, the DNAJC3‐AS1 gene sequence was sub‐cloned into the lentiviral expression vector pEZ‐Lv206 (GeneCopoeia, Guangzhou, China) for up‐regulation; sh‐RNA of DNAJC3‐AS1 was sub‐cloned into vector psi‐LVRU6MP for gene silencing. The resulting construct of pEZ‐Lv206‐DNAJC3‐AS1 and psi‐LVRU6MP‐DNAJC3‐AS1 was verified by DNA sequencing. And the control groups are their respective empty vector. After constructed, the plasmid vector was stably transduced into OS cell lines. All sequences are listed in the Appendix S1.

### 2.6 Transient transfection

The siRNA of DNAJC3 was purchased from GenePharma (Suzhou, China) for down‐regulation, and the plasmid vector EX‐K0780‐M61 with DNAJC3 gene sequence was purchased from GeneCopoeia, Guangzhou, China for up‐regulation. Si‐DNAJC3 or up‐DNAJC3 and their respective control vector were transfected into OS stable transfection cell lines, respectively, using Lipofectamine 3000 reagent (Gibco, Life Technologies) according to the manufacturer's protocol. All sequences are listed in the Appendix S1.

### 2.7 Cell proliferation assay

A total of 1000 transfected cells were seeded into 96‐well plates, respectively. The cell proliferation was determined at 12, 24, 36, 48, and 60 hours after incubated in 10% Cell Counting Kit‐8 (CCK‐8, Corning Corporation, Corning, NY, USA) at 37°C for 3 hours. OD value at 450 nm was detected using microplate reader. Each assay was performed in triplicate.

### 2.8 Plate clone formation assay

One hundred transfected were seeded into 6‐well plates and incubated for 30 days at 37°C. And then, we fixed cells with 4% paraformaldehyde for 30 minutes. After fixed, the cell colonies were stained with 0.1% crystal violet for 15 minutes. The number of colonies was counted with a scanner, after counted, the plate clone formation efficiency was calculated (plate clone formation efficiency = number of colonies/number of cells inoculated × 100%). The experiment was performed in triplicate.

### 2.9 Soft agar colony formation assay

Two milliliter 0.6% agar was layered in bottom onto 6‐well plates, followed by 2 mL 0.3% agar containing 1000 transfected cells as the top layer. Then, the cells were incubated for 4 weeks at 37°C, 5% CO_2_. After incubated, the cell colonies were stained with 0.1% crystal violet for 15 minutes. Cell colonies were counted and photographed under a microscope. These experiments were performed in triplicate.

### 2.10 Wound healing assay

Transfected cells were planted into 6‐well plates and grown to confluence. Then, the monolayer cells would be scratched manually with a sterile 200 μL pipette tip ensuring that the width of each scratch was consistent, and wounded monolayer cell was cultured for 24 hours in serum‐free medium at 37°C 5% CO_2_. Photographs of the central of wound edges would be taken at predicted stages (0 and 24 hours) after scratched by digital camera. The capacity of cell migration was quantified by analyzing the width of wound edges. And this assay was performed in triplicate.

### 2.11 Transwell invasion and migration assays

We performed cell transwell invasion and migration assays using 24‐well BD BioCoat Matrigel Invasion Chambers (8 μm pore size; BD Biosciences, SanJose, CA, USA). 1 × 10^5^ serum starvation OS cells were re‐suspended in 200 μL serum‐free medium and added to the upper wells of BD BioCoat Matrigel Invasion Chambers for invasion (with matrigel basement membrane matrix over the PET membrane) and migration (without matrigel basement membrane matrix). MEM (400 μL) containing 10% FBS was filled into the bottom chambers. After 24 hours of incubation, the cells were fixed with 4% paraformaldehyde for 30 minutes and stained with 0.1% crystal violet for 15 minutes. And then, the nonmigrated cells were wiped off from the surface of PET membrane with swab, the cells that migrate through the pores will be counted under the microscope and photographed in ten randomly selected fields. All the assays were performed in triplicate.

### 2.12 Detection of cell cycle and apoptosis by flow cytometry

After transfection, OS cells cycle analysis was determined by flow cytometry using Cell Cycle Analysis Kit (Biyuntian, China) according to the manufacturer's protocol. For apoptosis analysis, cells were treated with FITC‐Annexin V and propidium iodide (PI) in the dark, and then cells were analyzed by flow cytometry according to the manufacturer's guidelines. All experiments were performed in triplicate.

### 2.13 Anti‐cancer drug sensitivity test

Transfected cells were seeded into 96‐well plates at the density of 1000 cells per well, cisplatin solution was added into the wells at the final concentration gradient of 0, 2, 4, 8, 16, 32, 64, and 128 μg/mL, after incubated for 24 hours at 37°C, the optical density at 450 nm was detected after cells were incubated in 10% CCK‐8 for 2 hours, and concentration of the half maximal inhibitory concentration (IC50) was calculated. All the assays were performed in triplicate.

### 2.14 Western blotting analysis

Transfected cells were lysed using 1 × RIPA buffer. Identical quantities of proteins were separated by 10% SDS‐polyacrylamide gels and electro‐transferred to PVDF nitrocellulose membranes. The membranes were then blocked by blocking buffer and incubated overnight, respectively, with antibodies for DNAJC3 (Abcam) or GAPDH at 4°C. After twice washes, the membranes were incubated, respectively, with goat anti‐rabbit secondary antibody at 37°C for 2 hours and the brands were analyzed with an enhanced chemiluminescence system.

### 2.15 Tumor xenograft assay

About 400 μL cell suspension solution containing 2 × 10^7^ cells were injected subcutaneously into the scruff of male BALB/C‐nu mice (4 mice in each group) purchased from Nanjing University. After housed for 1 week, the tumor mass was examined every 3 days, the tumor mass was analyzed by measuring tumor length (*L*) and width (*W*), and tumor volumes were calculated according to the equation *V* = 0.5 × *LW* ^2^. Tumor weights were weighed by electronic scale. After that tumor nodules were fixed with 4% paraformaldehyde for immunohistochemistry and TUNEL assay.

For the tail vein transfer experiment, about 400 μL cell suspension solution containing 2 × 10^7^ cells were injected into the body of nude mice (4 mice in each group) through tail vein. Eight weeks after injection, all the mice were sacrificed; the lungs were taken out and photographed, following by making into slides and hematoxylin‐eosin staining. All procedures for animal care were complied with ethical standards and approved by the Animal Management Committee of The Southern Medical University.

### 2.16 Immunohistochemistry

Tumor nodule was fixed with 4% paraformaldehyde, embedded in paraffin, and cut into 4 μm‐thick section. After deparaffinized, rehydrated, and antigen repaired, the sections were treated with 3% hydrogen peroxide solution to quench endogenous peroxidase activity. After washed and blocked, Polyclonal rabbit antibody against Ki‐67 (Abcam, Cambridge, MA, USA) was added and incubated at 4°C overnight. After washing, sections were incubated with specific antibodies horseradish peroxidase (HRP)‐conjugated secondary antibodies for 2 hours at room temperature. Then, treated with DAB and counterstained with hematoxylin. Sections were sealed with Neutral balsam and analyzed by optical microscopy.

The lungs collected form nude mice were fixed with 4% paraformaldehyde, embedded in paraffin, and cut into 4 μm‐thick section. After HE stained, the sections mounted with xylene‐based mounting medium and photographed with a light microscopy.

### 2.17 TUNEL assay

The tumor tissues were collected from nude mouse and fixed with 4% paraformaldehyde for making slides. TUNEL assays were performed by using DeadEnd Fluorometric TUNEL System (G3250, Promega, America) according to the manufacturer's protocol. After that all the slides were detected the localized green fluorescence of apoptotic tissue and photographed by using AXIO‐Scope.A1 (ZEISS, Germany).

### 2.18 Statistics

All statistical analyses were performed using the SPSS17.0 (SPSS, Chicago, IL, USA). The significance of differences between OS specimens and matched normal tissues was estimated using paired samples *t* test, and Pearson's coefficient correlation was used to analyze the relationship between *DNAJC3‐AS1* and *DNAJC3*. Others comparisons were analyzed by chi‐square test or analysis of variance (ANOVA). *P* < 0.05 was considered statistically significant.

## 3 RESULTS

### 3.1 DNAJC3‐AS1 expression is up‐regulated in OS specimens and cell lines

**Figure 1:**
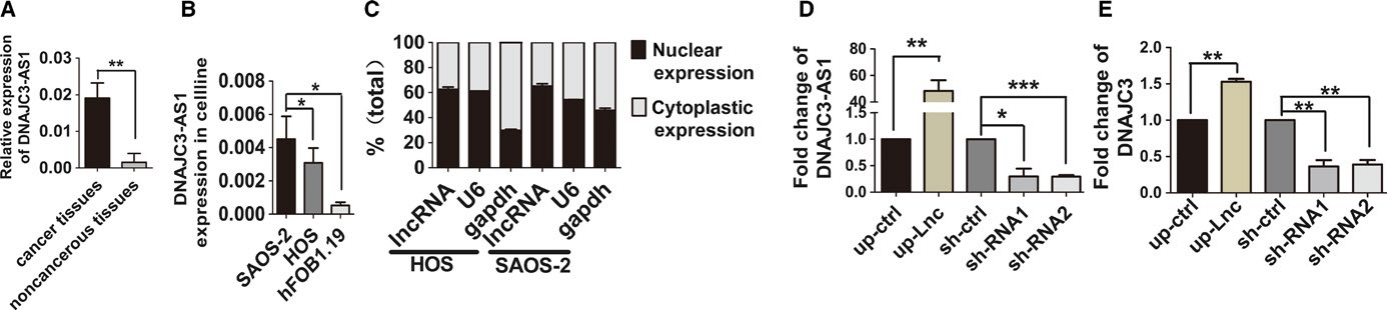
Osteosarcoma specimens and cell lines exhibit higher *DNAJC3‐AS1* expression level. A, The expression of *DNAJC3‐AS1* in OS specimens (n = 30) was compared with the pair‐matched noncancerous specimens (n = 30). B, The expression level of *DNAJC3‐AS1* in HOS and SAOS‐2 were compared with hFOB1.19. C, The subcellular location of *DNAJC3‐AS1* was identified using qRT‐PCR in HOS cells and SAOS‐2 cells. D, Fold change of DNAJC3‐AS1 in stable transfected HOS cells detecting by qRT‐PCR analysis. E, Fold change of DNAJC3 in stable transfected HOS cells detecting by qRT‐PCR analysis. We define up‐regulated LncRNA DNAJC3‐AS1 as up‐Lnc, down‐regulated LncRNA DNAJC3‐AS1 as sh‐RNA1 or 2 and their respective control group as up‐ctrl and sh‐ctrl. Data were expressed as the mean ± SD. *P < 0.05, **P < 0.01, ***P < 0.001

Expression of DNAJC3‐AS1 was examined in 30 pairs of OS specimens and pair‐matched adjacent noncancerous tissues by using qRT‐PCR. As shown in Figure 1A, DNAJC3‐AS1 exhibited increased expression in OS tissues compared with their pair‐matched adjacent noncancerous tissues (*P *< 0.01). Moreover, expression of DNAJC3‐AS1 was also up‐regulated in OS cell lines, SAOS‐2, and HOS, as compared with hFOB1.19 (Figure 1B). We also assessed the subcellular location of DNAJC3‐AS1. Subcellular fraction analysis revealed that DNAJC3*‐AS1* was mainly localized in cytoblast rather than cytoplasm, suggesting DNAJC3‐AS1 as a transcriptional regulation factors in OS (Figure 1C).

### 3.2 DNAJC3‐AS1 correlates with clinical features of OS and patients’ prognosis

To access the correlation between DNAJC3‐AS1 and clinicopathologic characteristics, the OS specimens were classified into high DNAJC3‐AS1 group (n = 24) and low DNAJC3‐AS1 group (n = 6) on the basis of the median DNAJC3‐AS1 expression level of all specimens. As shown in Table 1 (Fisher's exact test for *P* value), high DNAJC3‐AS1 expression was related to high differentiated degree and advanced Enneking stage of OS by correlation regression analysis. These results indicated DNAJC3‐AS1 played positive role in OS development and progression.

**Table 1:** The association between clinicopathological characteristics and the expression of *DNAJC3‐AS1*

Factors	Amount of patient (n = 30)	*DNAJC3‐AS1* expression		*P* ^a^ value
		Low (n = 6)	High (n = 24)	
Age				
≤20	8	2	6	0.645
20	22	4	18	
Gender				
Male	20	5	15	0.663
Female	10	1	9	
Location				
Tibia/femur	24	3	21	0.075
Elsewhere	6	3	3	
Histological type				
Osteoblastoma	19	2	17	0.156
Else	11	4	7	
Differentiated degree				
High/middle	4	3	1	**0.018**
Low/undifferentiation	26	3	23	
TNM				
T1N0M0	7	2	5	0.603
T2N0M0	23	4	19	
Clinical stage				
I	12	5 (41.7)	7 (58.3)	**0.016**
II	18	1 (5.6)	17 (94.4)	

DNAJC3‐AS1 expression level was examined using qRT‐PCR, and low or high *DNAJC3‐AS1* expression group was classified by the median expression of all specimens.

*P* value (<0.05) was shown in bold type.

Fisher’s Exact Test *P* value

### 3.3 DNAJC3‐AS1 facilitates the malignant biological behaviors of OS cells in vitro

**Figure 2:**
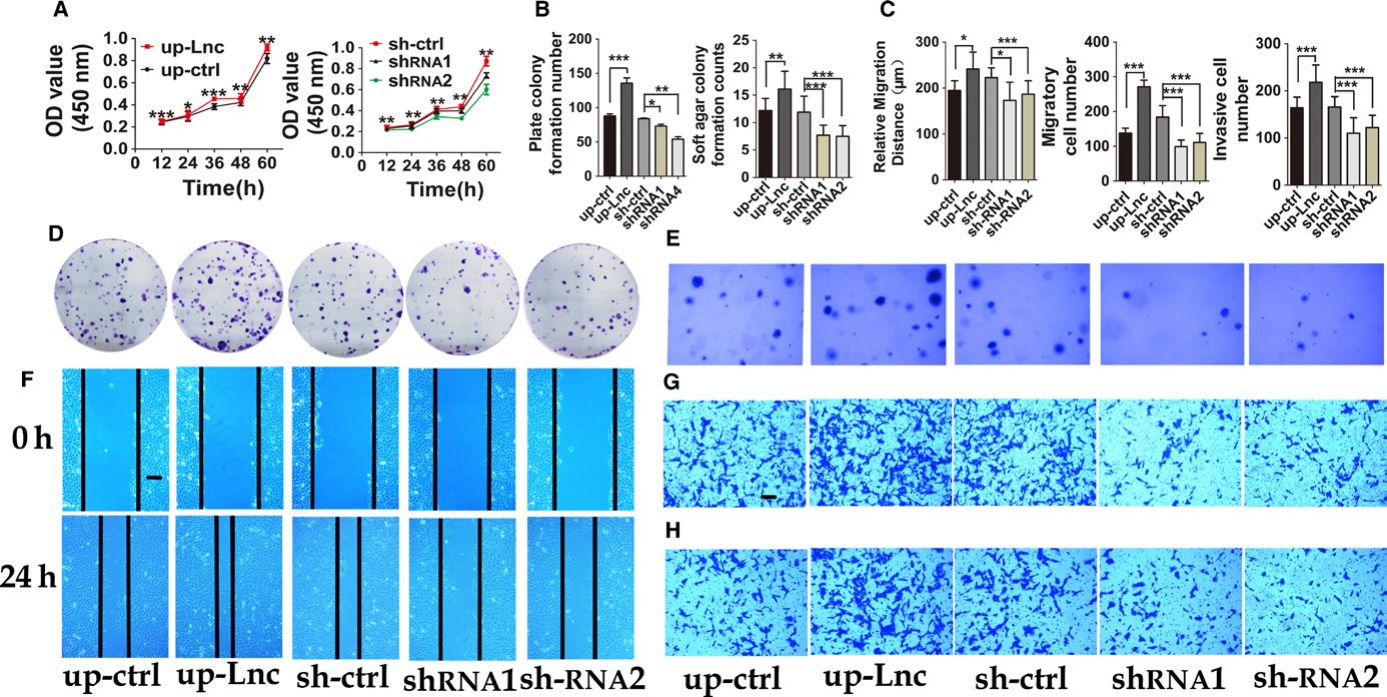
*DNAJC3‐AS1* promotes cells proliferation, migration, and invasion capacity of HOS cells (A) CCK‐8 assay, (B (left) and D) Clone formation and (B(right) and E) Soft agar clone formation showed that down‐regulated DNAJC3‐AS1 suppressed proliferation of HOS cells, and up‐regulated DNAJC3‐AS1 did the opposite. (F and C (left)) wound healing assay and (G and C (middle)) migration assay showed that up‐regulated DNAJC3‐AS1 improved migration ability of HOS cells, and down‐regulated DNAJC3‐AS1 did the opposite. (H and C (right)) Invasion assay showed that DNAJC3‐AS1 improved invasion capacity of HOS cells. All the photographs were randomly selected and taken at ×100 field. Scale bar, 200 μm. Data were expressed as the mean ± SD. The results were reproducible in three independent experiments. *P < 0.05, **P < 0.01, ***P < 0.001

**Figure 3:**
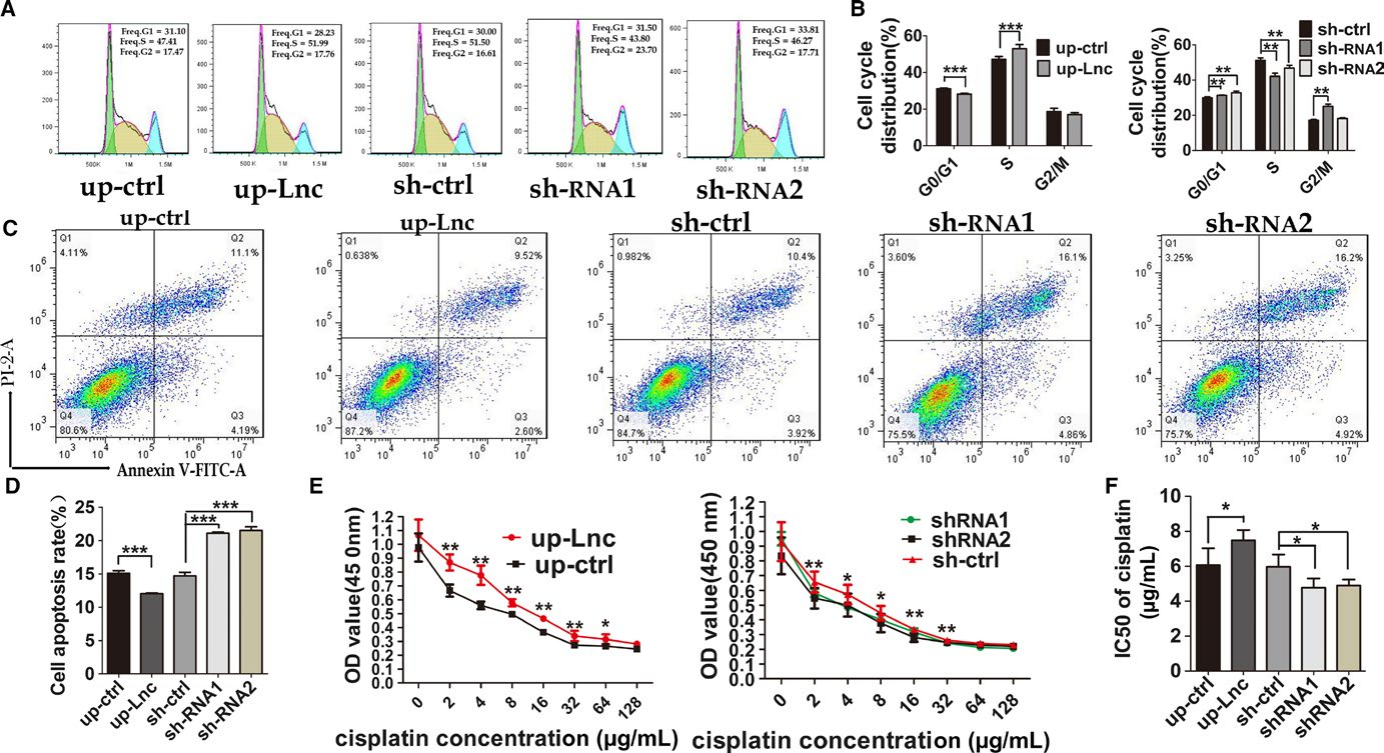
*DNAJC3‐AS1* not only promotes HOS cells proliferation, but also inhibits HOS cells apoptosis and increases drug resistance to cisplatin of HOS cells. A and B, Flow cytometer analysis indicated that up‐regulated DNAJC3‐AS1 increased the percentage of S phase cells and decreased the percentage of G0/G1 phase cells, and down‐regulated DNAJC3‐AS1 did the opposite. C and D, Cell apoptosis assay showed that down‐regulated DNAJC3‐AS1 promoted HOS cells apoptosis rate. And up‐regulated DNAJC3‐AS1 reduced HOS cells apoptosis rate. E, CCK8 assay showed that up‐regulated DNAJC3‐AS1 reduces sensitivity of HOS cells to cisplatin, and down‐regulated DNAJC3‐AS1 does the opposite. F, The IC50 of HOS cells with up‐regulated or down‐regulated DNAJC3‐AS1 and their respective control groups. Data were expressed as the mean ± SD. The results were reproducible in three independent experiments. *P < 0.05, **P < 0.01, ***P < 0.001

To prove the positive function of DNAJC3‐AS1 in vitro, we firstly up‐regulated or disturbed DNAJC3‐AS1 expression level in OS cells (Figure 1D and Figure S1B), and these changes significantly resulted in decrease or increase of DNAJC3 mRNA, respectively (Figures 1E and S1C). And then, we investigated the roles of DNAJC3‐AS1 in OS cells. We detected the proliferative rate of OS stable transfected cells with DNAJC3‐AS1 up‐ or down‐regulated using CCK‐8 assay. The results revealed that DNAJC3‐AS1 promoted proliferation of OS cells and depletion of DNAJC3‐AS1 significantly suppressed cell proliferation (Figures 2A and S2A). These results were further confirmed in colony formation assay and soft agar colony formation assay (Figures 2D,E and S2D,E), and the statistic analysis was shown in Figures 2B and S2B. In wound healing and migration assay, OS cells with elevated DNAJC3‐AS1 migrated faster than their control, while cells with decreased lncRNA showed opposite effect on cell migration (Figures 2F,G and S2F,G), and the statistic analysis was shown in Figure 2C (left and middle) and S2C (left and middle). As shown in Figures 2H and S2H, up‐regulation of DNAJC3‐AS1 promoted OS cell invasion, while transfection of cells with sh‐DNAJC3‐AS1 impeded cell invasion ability, and the statistic analysis was shown in Figures 2C (right) and S2C (right). Mechanisms for the positive role of DNAJC3‐AS1 in cell proliferation were uncovered by flow cytometry analysis, results from which revealed that the lncRNA‐DNAJC3‐AS1 decreased OS cells in G0/G1 phase and increased the number in S phase (Figures 3A,B and S3A,B). Effect of DNAJC3‐AS1 on OS cell apoptosis was also examined by using flow cytometry. Up‐regulation of the lncRNA reduced apoptosis rate of OS cells, while OS cells interfered with DNAJC3‐AS1 expression showed elevated apoptosis rate (Figures 3C,D and S3C,D).

### 3.4 DNAJC3‐AS1 reduces osteosarcoma drug resistance

Chemotherapeutic agent resistance comes not only from individual differences of patients, but also from genetic and epigenetic differences of tumors. We then investigated the function of DNAJC3‐AS1 affects sensitivity of OS cells to cisplatin. Concentration gradient of cisplatin resulted in concentration‐dependent death of OS cells. Up‐regulated DNAJC3‐AS1 level led to drug resistance of OS cells to cisplatin, while down‐regulating DNAJC3‐AS1 accelerated death of the cells (Figures 3E and S3E). As expected, the IC50 for cisplatin was increased when DNAJC3‐AS1 was up‐regulated. On the contrary, the IC50 was decreased when the lncRNA was down‐regulated (Figures 3F and S3F). These results indicated that DNAJC3‐AS1 impairs the sensitivity of OS cells to cisplatin.

### 3.5 DNAJC3‐AS1 promotes xenograft OS growth and metastasis in mice

**Figure 4:**
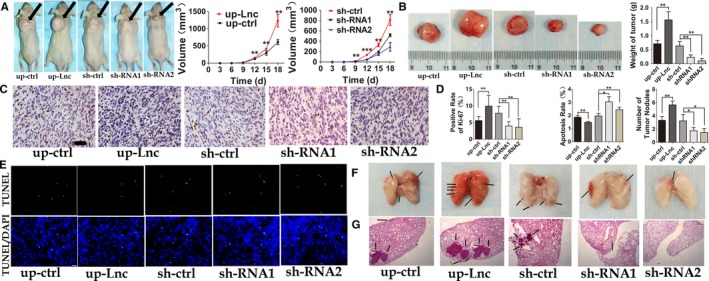
DNAJC3‐AS1 promotes OS growth and inhibits cells apoptosis and accelerates distant metastasis of OS cells in vivo. A, Representative photographs taking at 28th day after injecting transfected HOS cells subcutaneously into the scruff of male BALB/C‐nu mice (arrows show the tumor nodules), and the statistic showed that DNAJC3‐AS1 increased the growth speed of tumor nodules in vivo significantly more than in the control group, and down‐regulated DNAJC3‐AS1 did the opposite. B, Representative images and the average weight of tumor nodules collected from each group at 28th day after injection. C, Immunohistochemical staining of Ki‐67 (x400; scale bar: 100 μm) and (D left) statistic analysis of Ki‐67 positive staining cells rate indicated that DNAJC3‐AS1 promotes OS cells proliferation in vivo. E, Emblematic figures of TUNEL assay (400 × , scale bar: 100 μm) and (D middle) statistic analysis of TUNEL‐positive staining cells rate indicated that DNAJC3‐AS1 promotes OS cells proliferation in vivo. F, Emblematic photographs of nude mice lung taking at 28th day after injected transfected HOS cells via tail vein. And (D right) the statistics of tumor nodules number which appeared on the surface of lung (arrows show the tumor nodules). G, The microscopic sections of lung tissues with HE stain, the metastatic tumor was shown as indicated by the arrows. Data were expressed as the mean ± SD. *P < 0.05, **P < 0.01, ***P < 0.001

To prove whether there was the same effect in vitro as the results showed above in vivo, we injected HOS cells (up‐DNAJC3‐AS1 OR down‐DNAJC3‐AS1) into nude mice. Up‐regulated DNAJC3‐AS1 caused tumor to grow faster than control group, while down‐regulated DNAJC3‐AS1 resulting in slowing down tumor growth. (Figure 4A,B). To determine whether DNAJC3‐AS1 affect the cell proliferation and apoptosis of OS in vivo or not, we executed Ki‐67 and TUNEL assay. As shown in Figure 4C,D (left), up‐regulating DNAJC3‐AS1 expression resulted in higher Ki‐67 positive rate indicating higher proliferation rate of OS cells, while down‐regulating DNAJC3‐AS1 expression inhibited proliferation of cells in vivo. For TUNEL assay, DNAJC3‐AS1 decreased the apoptosis rate of OS cells in vivo (Figure 4E,D (middle)). Moreover, we also injected nude mice with HOS cells through their tail vein and monitored tumor metastasis. OS cells with up‐regulated DNAJC3‐AS1 resulted in more lung metastasis in mice, and the metastatic tumor nodules were shown as indicated by the arrows (Figure 4F, D (right), and G).

### 3.6 DNAJC3‐AS1 associates positively with sense‐cognate gene *DNAJC3* in OS cells

**Figure 5:**
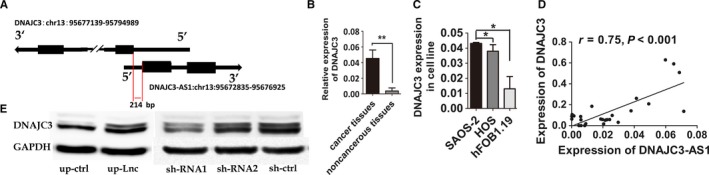
DNAJC3‐AS1 associates positively with its sense‐cognate gene DNAJC3 in HOS cells. A, Schematic diagram of the structure of DNAJC3‐AS1 and DNAJC3. Arrows indicate the transcription direction of gene, and blocks are representative of exons. B, The expression of *DNAJC3* in OS specimens (n = 30) was compared with the pair‐matched noncancerous specimens (n = 30). C, The expression level of *DNAJC3* in HOS and SAOS‐2 were compared with hFOB1.19. D, Correlation analysis revealed apparent positive relationship between DNAJC3‐AS1 and DNAJC3 in OS specimens (*r* = 0.75, *P < 0.001*). E, Western blot analysis of *DNAJC3* in HOS cells with down or up‐regulating *DNAJC3‐AS1*. Data were expressed as the mean ± SD. The results were reproducible in three independent experiments. *P < 0.05, **P < 0.01, ***P < 0.001

In order to investigate internal regulation mechanism of DNAJC3‐AS1, we then performed biological information analysis, which showed that DNAJC3‐AS1 and DNAJC3 constituted a “head‐to‐head” pairing pattern with DNAJC3‐AS1 overlapping the promoter region of DNAJC3 completely (Figure 5A). Therefore, we detected the correlation between DNAJC3‐AS1 and DNAJC3. We observed an increasing expression of DNAJC3 level in OS specimens compared with their corresponding noncancerous specimens (Figure 5B). Furthermore, OS cell lines also presented higher expression level of DNAJC3 compared with hFOB1.19 cells (Figure 5C). Importantly, correlation analysis revealed a positive relationship between DNAJC3‐AS1 and DNAJC3 expression level over OS specimens (Figure 5D). These results were further more supported by Western blotting analysis (Figures 5E and S1A). These results demonstrated that DNAJC3‐AS1 may participate in the development and progression of osteosarcoma via regulating its sense‐cognate gene DNAJC3, indicating DNAJC3 as a possible mediator of biological function of DNAJC3‐AS1.

### 3.7 DNAJC3‐AS1 accelerates osteosarcoma progression via up‐regulating DNAJC3

**Figure 6:**
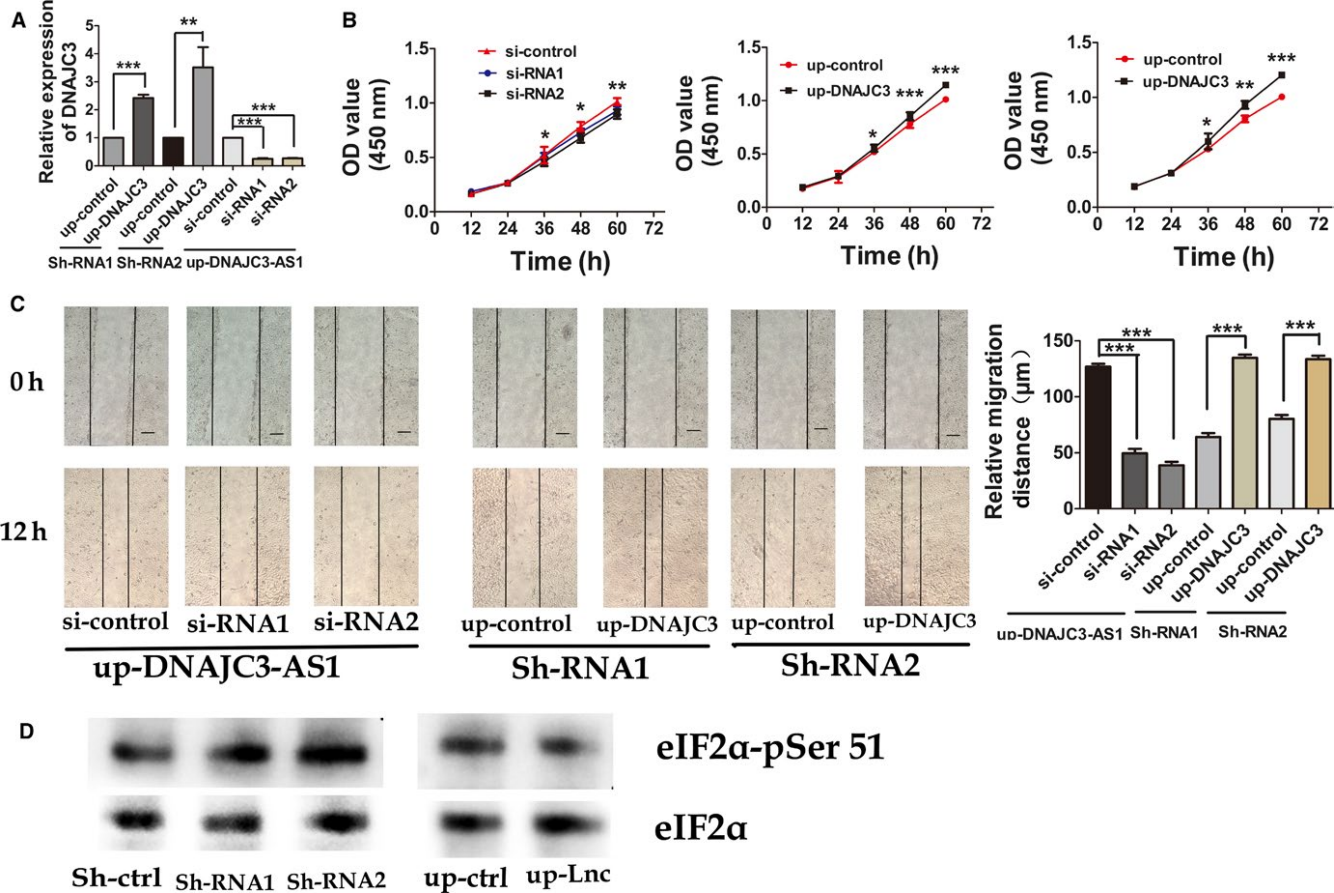
DNAJC3‐AS1 accelerates osteosarcoma progression via DNAJC3. A, HOS cells with stably down‐regulated (Sh‐RNA1 and Sh‐RNA2) or up‐regulated DNAJC3‐AS1 (up‐DNAJC3‐AS1) were over‐expressed with DNAJC3 (up‐DNAJC3) or interfered with DNAJC3 siRNA (si‐RNA1 and si‐RNA2). DNAJC3 expression in these cell sets was detected by RT‐PCR. B, Proliferation of the cell sets depicted in (A) was determined by CCK‐8 assay. C, Migration capacity of the cell sets was determined by wound healing assay. D, Western blot analysis of eIF2α and eIF2α‐pSer 51 in HOS cells with down‐regulated or up‐regulated *DNAJC3‐AS1*. Data were expressed as the mean ± SD, all the images are 400×, scale bar, 100 μm. The results were reproducible in three independent experiments. *P < 0.05, **P < 0.01, ***P < 0.001

Furthermore, we did the following to convince the potential function of DNAJC3‐AS1 at regulating the development and progression of osteosarcoma through adjusting the expression of DNAJC3. As had been shown above, up‐regulating DNAJC3‐AS1 expression could induce cell proliferation (CCK 8 assay) and migration (wound healing assay) of osteosarcoma, while down‐regulating DNAJC3‐AS1 expression did the opposite. However, both the effects were reversed by DNAJC3 down‐ or up‐regulation, respectively (Figure 6B,C and Figure S4B,C). What`s more, it has been reported that DNAJC3 can reduce the phosphorylation of eIF2α, which results in the decrease of cell apoptosis rate. Down‐regulating DNAJC3‐AS1 led to the increase of eIF2α phosphorylation at serine 51 in OS cells (Figures 6D and S4D). These proofs proved that DNAJC3‐AS1 functioned in osteosarcoma cells by adjusting the expression of DNAJC3 positively.

## 4 DISCUSSIONS

Owing to their various functions in the pathogenesis of diseases, lncRNAs have been widely studied in different types of tumors.^3^, ^8^, ^18^, ^29^, ^30^, ^31^, ^32^ Among the diverse kinds of lncRNAs, antisense lncRNAs attract more and more investigations. Such as, HNF1A‐AS1 has been found to promote the progression of OS via regulating the Wnt/β‐catenin pathway, indicating HNF1A‐AS1 as a potential target for the treatment of OS. Knockdown of FGFR3‐AS1 inhibits OS cells proliferation and cell cycle progression in vitro and inhibits xenograft tumor growth of OS cells in vivo.^1^ In addition, HOXD‐AS1/miR‐130a sponge regulates glioma development by targeting E2F8.^33^

In our study, we observed that DNAJC3‐AS1 is up‐regulated in OS specimens compared with adjacent noncancerous specimens. And high DNAJC3‐AS1 expression is correlated with low differentiated degree, metastasis, and poor prognosis. Furthermore, we uncovered the effects of DNAJC3‐AS1 in OS cells in vitro and in vivo. These experiments revealed that DNAJC3‐AS1 promoted cell proliferation, invasion, and migration and inhibited cell apoptosis of OS in vitro*,* and as well as accelerated tumor growth in vivo. All the results indicated DNAJC3‐AS1 was a carcinogene in OS.

Besides, not only DNAJC3‐AS1 correlated positively with DNAJC3, but also all these effects of DNAJC3‐AS1 were reversed by *DNAJC3* up‐ or down‐regulated, which indicated DNAJC3‐AS1 might exerts its function in OS cells via its sense‐cognate gene DNAJC3, which has been reported to be involved in cell adaptive damage or apoptosis and cancer's development and progression, such as prostate cancer and breast cancer.^21^, ^24^, ^25^, ^26^ Mechanistically, down‐regulated DNAJC3 can induce the phosphorylation of eIF2α, which thing could accelerate cell apoptosis via endoplasmic reticulum apoptosis pathway.^34^, ^35^, ^36^

Treatments for OS relay on surgical resection of the tumor bulk combined with chemotherapy and/or radiotherapy, which significantly improve the five‐year survival rate of OS patients.^37^, ^38^, ^39^ However, the frequency of recurrence and chemotherapy resistance decreased survival time of patients.^38^, ^40^, ^41^, ^42^ In the present study, we uncovered that DNAJC3‐AS1 decreased the chemotherapeutic drug sensitivity of OS cells to cisplatin obviously. Added to the role of DNAJC3‐AS1 in proliferation, migration, invasion, and apoptosis of OS cells, DNAJC3‐AS1 is a potential therapeutic target for OS.

Collectively, we here uncover that DNAJC3‐AS1 is up‐regulated in OS tissues and its high expression is associated with poor prognosis of OS patients. Down‐regulating DNAJC3‐AS1 expression suppresses OS cells growth in vitro and in vivo. DNAJC3‐AS1 promotes OS growth and development via up‐regulating DNAJC3 to reduce the phosphorylation of Eif2α which thing induces to cell apoptosis by endoplasmic reticulum apoptosis pathway. These findings reveal that DNAJC3‐AS1 could be a potential biomarker for prognosis evaluation and therapeutic target for OS.

## CONFLICT OF INTEREST

None declared.
