# Supplementary figures and images for: Long noncoding RNA DNAJC3‐AS1 promotes osteosarcoma progression via its sense‐cognate gene DNAJC3
Source: Cancer Med. 2019 Jan 16;8(2):761–72. doi: 10.1002/cam4.1955 (PMC6382712; doi:10.1002/cam4.1955)

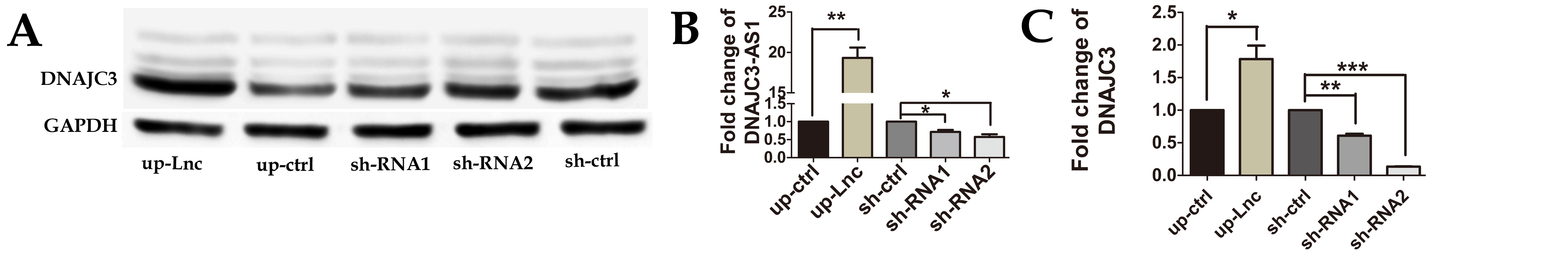

Supplement: Supplementary file 1 [file CAM4-8-761-s001.tif]

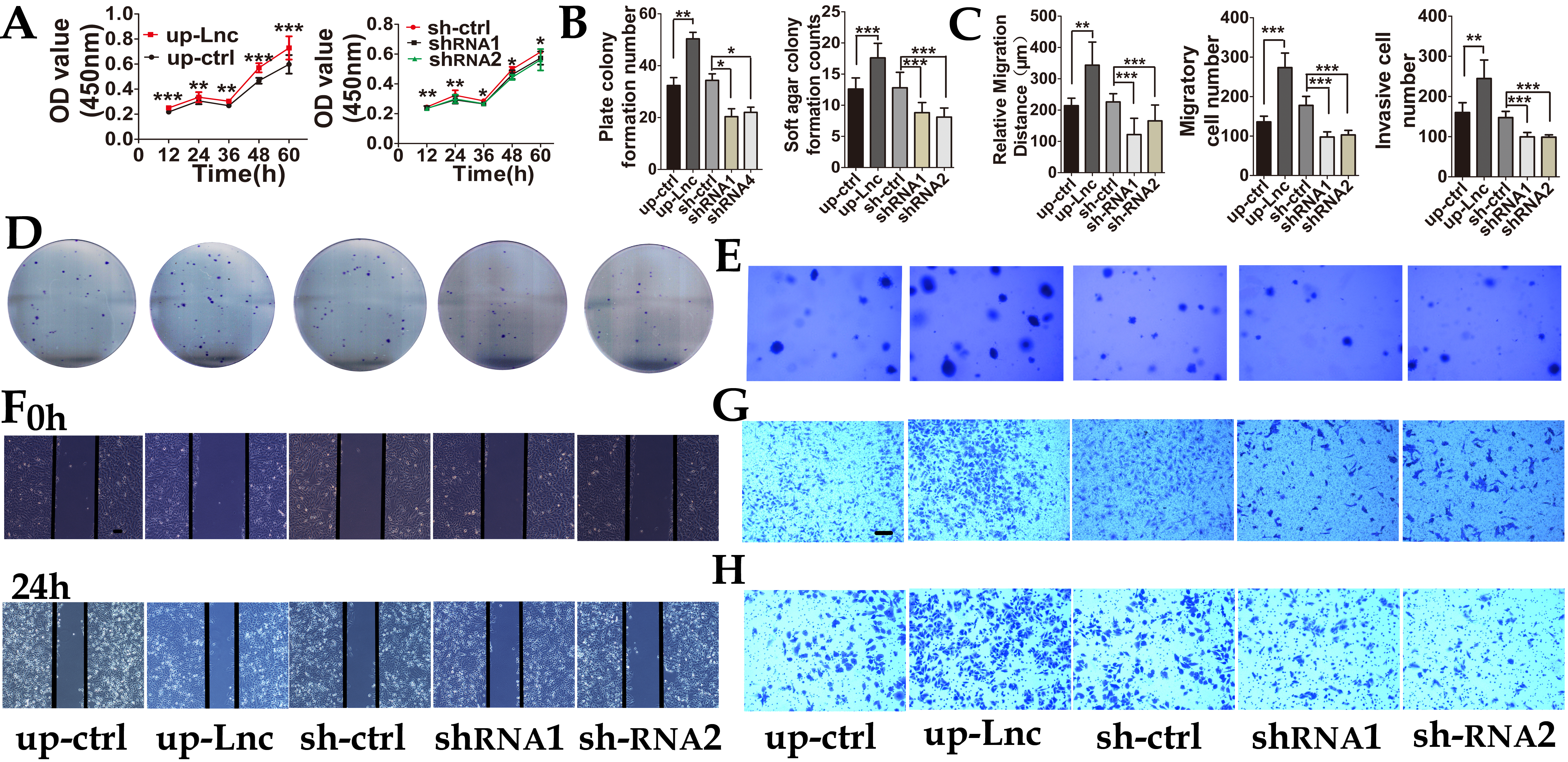

Supplement: Supplementary file 2 [file CAM4-8-761-s002.tif]

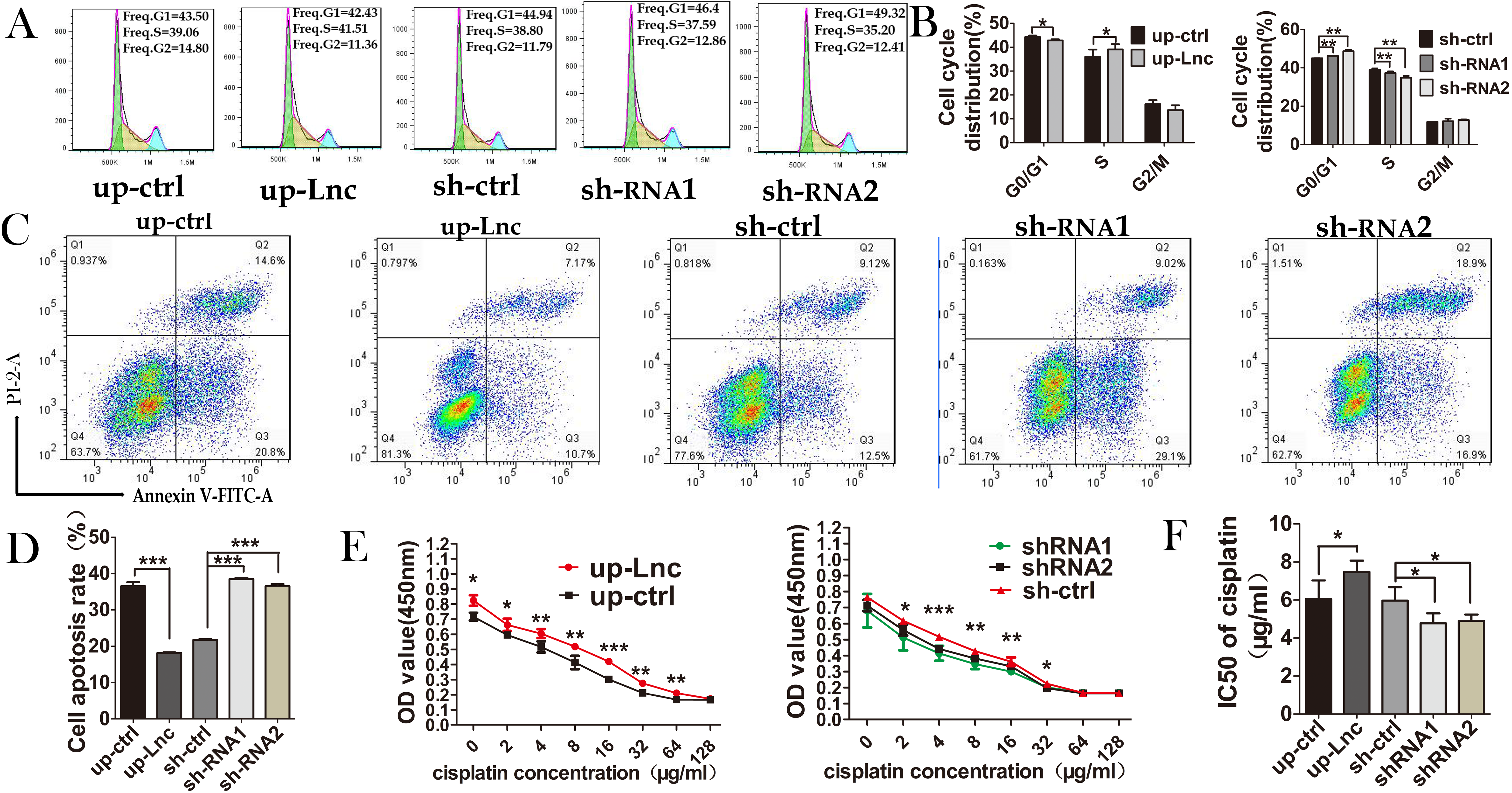

Supplement: Supplementary file 3 [file CAM4-8-761-s003.tif]

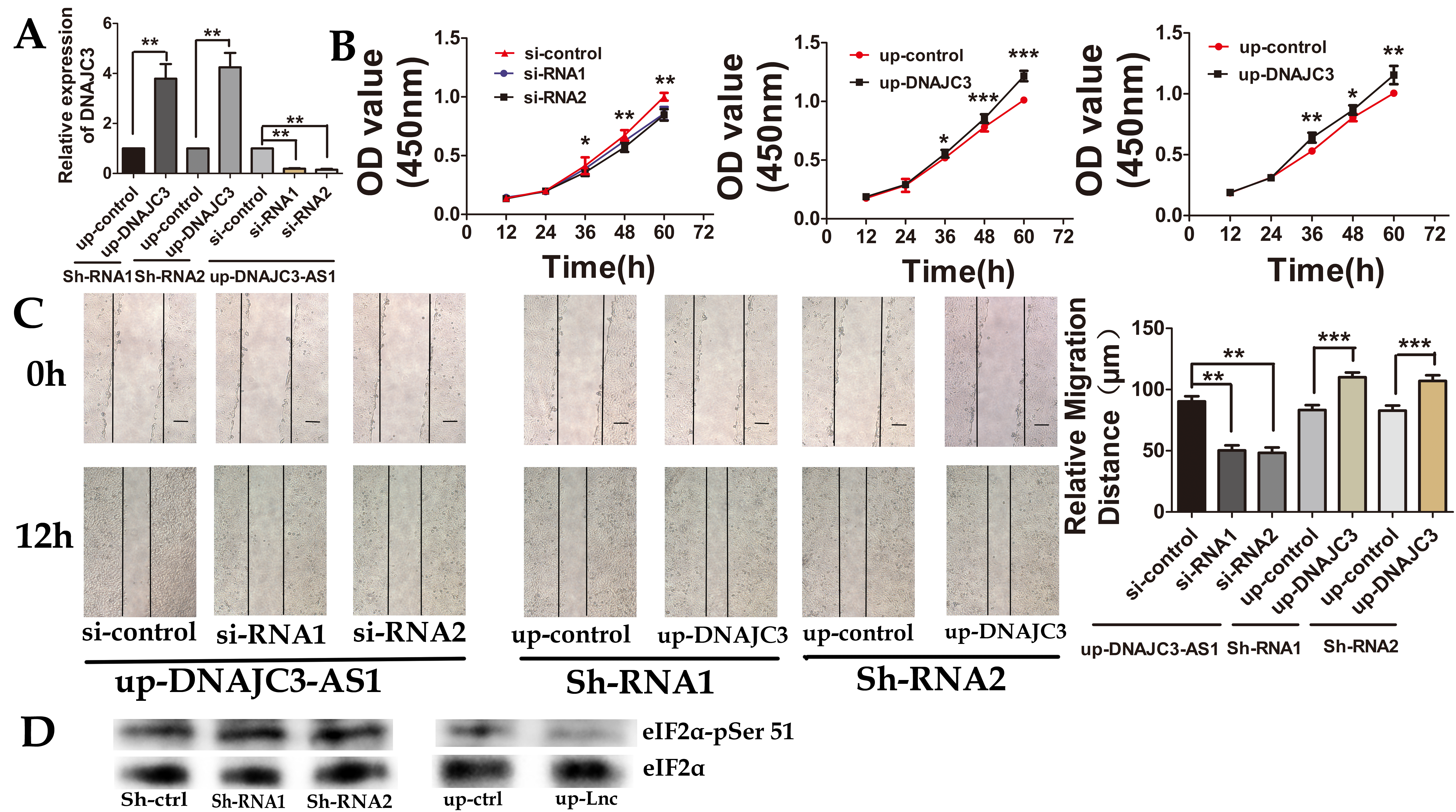

Supplement: Supplementary file 4 [file CAM4-8-761-s004.tif]
