# Supplementary material for: Long noncoding RNA DNAJC3‐AS1 promotes osteosarcoma progression via its sense‐cognate gene DNAJC3
Source: Cancer Med. 2019 Jan 16;8(2):761–72. doi: 10.1002/cam4.1955 (PMC6382712; doi:10.1002/cam4.1955)
Supplement: Supplementary file 5 [file CAM4-8-761-s005.docx]

**Figure Legends:**

**Fig.S1 lnc-*DNAJC3-AS1* correlates positively with its sense-cognate gene *DNAJC3* in SAOS-2 cells.** **(A)** Western blot analysis of *DNAJC3* in SAOS-2 cells with down or up regulating *DNAJC3-AS1.* (B) Fold change of DNAJC3-AS1 in stable transfected SAOS-2 cells detecting by qRT-PCR analysis. (C) Fold change of DNAJC3 in stable transfected SAOS-2 cells detecting by qRT-PCR analysis. Data was expressed as the mean ± SD. The results were reproducible in three independent experiments. **P < 0.05, ** P < 0.01, *** P < 0.001*.

**Figure S2 *DNAJC3-AS1* promotes cells proliferation, migration and invasion capacity of SAOS-2 cells. (A)** CCK-8 assay, (B （left）and D) Clone formation and (B(right) and E) Soft agar clone formation showed that down-regulated DNAJC3-AS1 suppressed proliferation of SAOS-2 cells, and up-regulated DNAJC3-AS1 did the opposite. **(F and C (left))** wound-healing assay and **(G and C (middle))** Migration assay showed that up-regulated DNAJC3-AS1 improved migration ability of SAOS-2 cells, and down-regulated DNAJC3-AS1 did the opposite. **(H and C (right))** Invasion assay showed that DNAJC3-AS1 improved invasion capacity of SAOS-2 cells*.* All the photographs were randomly selected and taken at ×100 field. Scale bar, 200μm. Data was expressed as the mean ± SD. The results were reproducible in three independent experiments. **P < 0.05, ** P < 0.01, *** P < 0.001*.

**Figure S3 *DNAJC3-AS1* not only promotes SAOS-2 cells proliferation, but also inhibits SAOS-2 cells apoptosis and increases drug resistance to cisplatin of SAOS-2 cells. (A and B)** Flow cytometer analysis indicated that up-regulated DNAJC3-AS1 increased the percentage of S phase cells and decreased the percentage of G0/G1 phase cells, and down-regulated DNAJC3-AS1 did the opposite. **(C and D)** Cell apoptosis assay showed that down-regulated DNAJC3-AS1 promoted SAOS-2 cells apoptosis rate. And up-regulated DNAJC3-AS1 reduced SAOS-2 cells apoptosis rate. **(E)** CCK8 assay showed that up-regulated DNAJC3-AS1 reduces sensitivity of SAOS-2 cells to cisplatin, and down-regulated DNAJC3-AS1 does the opposite. **(F)** The IC50 of SAOS-2 cells with up-regulated or down-regulated DNAJC3-AS1 and their respective control groups. Data was expressed as the mean ± SD. The results were reproducible in three independent experiments. **P < 0.05, ** P < 0.01, *** P < 0.001.*

**Figure S4 DNAJC3-AS1 accelerates osteosarcoma progression via DNAJC3 (A)** SAOS-2 cells with stably down-regulated (Sh-RNA1 and Sh-RNA2) or up-regulated DNAJC3-AS1 (up-DNAJC3-AS1) were over-expressed with DNAJC3 (up-DNAJC3) or interfered with DNAJC3 siRNA (si-RNA1, si-RNA2). DNAJC3 expression in these cell sets were detected by RT-PCR. **(B)** Proliferation of the cell sets depicted in (A) was determined by CCK-8 assay. **(C)**Migration capacity of the cell sets was determined by wound-healing assay*.* **(D)** Western blot analysis of eIF2α and eIF2α-pSer 51 in SAOS-2 cells with down-regulated or up-regulated *DNAJC3-AS1.* Data was expressed as the mean ± SD, all the images are 400×, scale bar, 100μm. The results were reproducible in three independent experiments. **P < 0.05, ** P < 0.01, *** P < 0.001*.

**Appendi S1: the sequences which are related to the study**

**Lnc-DNAJC3-AS1 over-expression**

5′-GAGCTGTCAGCAAGCTCCAAACCGCGGCTGCCGCCCGGAGGGAAGCAGACGGGGAGTCGGTGGCGGCCGCCGAGTCCGCATGCGCAGGCTGCAAAGGCCCTTTTGGGCCGGTCCCGCCGCGCTAGGTTGGCGAGTGGGACTATTTGATCCTTTCCAGGTTCGCCAGCGTGGTGTTACGAGAGGAAAGCGCAGCCGCAGCGTACGAATGGGAAGGCCAAACACGGTCCCGGGGAGTCTGAGGGAAGCTGCAGGGCGCCTGGAATTGGGGTCTCGAGTGAGCCGGTAAAAACCGCCTCCCGCTGAGCGTCCCGGAACAAATACTGTCTGAACTGAGCCGTAAGCAAGAAACGGTCAGGAGAAGCTGGGGCGATCTGATGATTAGAACATTTCAGGGACTACCAGAGAGAAGTGCGTAATTGCTCTAAGGCAGGTAGGTAATTGGCCTGGAAACTTGGGAACGCCCCGCCCCCCACCCCGACTTTCAGCAAACCACCGTGCCCAGGAAAAAGAGCGATTGTGGAAGACCCTGTGAAGAAGAGACCAGGAAGGCACTGTGGCTTAAGAGAGCAAGGCGTTCCTGAATGCAGCCAACATCAACTGGTAAACAAAAAACTGTGAGAACGGATCCTGAATCTTGCGCTTACCAGGGGAAATTCAGGAGAGATATGAACCTGAGCAAGATGAGGTTGAAGTTACTCAATGCGGCATTAAGCCTGTTCACGGCCACTTCCTCCGGCGTCACCAACACACACACTGTACACCAGCAGTATTTTTACACGGGGTATGTGTGTTAAGTACTATCAGGTGGATAATTATTACTTCACCTGTATTTGCTGCCTTGATACCATTAGAGAAACTCATGAATTATAAATAGCATAGTGAATTTGTGATTCCCTGAAGATATACTAAGGTGGACTTTTAAAAAGGCTTCTATTCCAAAGGGAACTAATATGGAGGAAATGGTTTCGAGAAAGAAAAAAGAAAAAACGGAGTCCTTGATTAGTTGTGGGGAAGGCAACATAATGTAATGTGAAGAATAAAGGAGTCAGATCCAGGAAACCCCATAACTATTATGAAACATTTCTAGGCTGGGCGCAGTGGCTCACACCTGTAATGCTAGCGCTTTGGGAAGCCAAGATGGGAGGATTATTTGAGGCCGGGAGTTTGCGACCAGCCTGAGCAACATAGCAGGATCCTGTCTCTGCAAAAAAATTTAAAACTAGCCAGGTGCGGTGGCTCGTGCCTGTTGCCCTAGCTACTCAGGGAGACTGAGGCAGAGGATTGCTTCAACCCAGGAGTTTGAGGTTGCAGTGAGCTAAGATCATGTCACTGCACTCCAGACTGAGCAACAGAGTGACACTTTATAAAAAAAAAAAAAAAAAAAACTAGGTTTTTGAACTTGCTTAATCAAAGAATTTGTTTAAACTTTTAAGATGCGTTGTCAGATTATCTTTCCCTAAAGGAATAATTTCTATTCCTATCAGCTGTTTATATTCCTGCCTAGTCACCATCACTAGATATAATTGATTTTCAGTTTTTGCCAATCTGAGGAACAAAAAATGACCCTTATATGTCAAATTTACAGTTTATTTTCAAGGATTTTGGGATTCTGATCAAATTTTGGTACCTTCATATAAAATTTGGCTTTTATATCACATCTTGTCTTCTCTGCTTTCCACCCTCTTTTATGTTGTTTTTTGGCTTCTGGCCGCATGACTATAATCTCCGCTTTTGTATTCACATCACCTTCTGTCATTTTTGACCTTGCCTCCGTCTTACAAGGATCCTTGTAATTAATTATATTGGGCCTGCTGAGATAATCCAGGATATTCTTCCTACTCAAGTTCCTCAATTTAATCACATCTGCAAAAACTGCCTTTTGCTATAGAACAATGACAGGAGATTAGAATGTAAACATATTTGGGGGACCGTTATTCAGCTTAACACAATACGTCCCCCTTCATCAGGTGGAGCTTATTTTCCCTCCTTCCTTGAGTGTGGGCTGGACTTAGTGACTAACTTCCAAAGAACAGAGTATGGAAAGGGAGGAGGAGAGTAACTTCATAGTACAGAAACCTGGAAACACTGTCTTGGCCAGGTGGTCAAAGTTAATATCATCAAGTCATGTTGATAGCATATACTCCCAATATACTGTGATGAGAAGGGCAATTCACCTCTGTGGTATTCTCAAAACCTATAACCCAATCTAGTCTAAACATGAAAAAAAAAAAATCAAACTAAAATTGAAGGACATTCTATAAAACACCTGATCAGTATTCCTCAAAACTATCAACGTCGTGGGGAACAAGGAAAGATTGAAATACTGTAACAGACCAGAGGAAACTAAGGAAACTTAATTGATGACTGAATGCAGTGTGCTGTGTTGAACTGGATCCTAGAGAAAATAGACATTAGTGGAAAAACTACTGAAATATGAATAGTCTCAAGTTTAATAGTAGTGTACCATTATTACTTTCTTAGTTTTGACAAATGTACCATGGTTATGCAAGATGCTAACAATACAGAAAAATAAGGGGTATTTGGGACCTCCCTGTATTATCCTTATGCCCTTACTTGAAAGTTGTTTCAGCAATAGAAGTTTATTTAAAATATTGTGAAGGGCCTGAGTATTTTTAAATCAACTGCAGTGACTCTCTTTTGTGTATTTGTGTACAGATGAGTTTTTCTGCATCGAATTTTATCTCCTTTCCTTGCAGAAGAAAGAGAATTAGCCCTTTACAGAGATAACAGTTGCCTAAGCCAGCTCTTTTCTCCTCTTTTTGATGTTCTTCTTTTTCTCTCAGTTTGAAAATTGTAGGTTATTTGAGTTTTTAAAATGTCTATTGCTACATACACATTCTAGTTATATTCTAGTCCAAACAATCTAGTAAAATATCTAAAAGAAAAA -3′

**DNAJC3 over-expression:**

5′-ATGGTGGCCCCCGGCTCCGTGACCAGCCGGCTGGGCTCGGTATTCCCCTTCCTGCTAGTCCTGGTGGATCTGCAGTACGAAGGTGCTGAATGTGGAGTAAATGCAGATGTTGAGAAACATCTTGAATTGGGCAAGAAATTACTTGCAGCTGGACAGCTAGCTGATGCTTTATCTCAGTTTCATGCTGCCGTAGATGGCGACCCTGATAACTATATTGCTTATTATCGGAGGGCTACTGTCTTTTTAGCTATGGGCAAATCAAAAGCTGCACTTCCTGATTTAACTAAAGTGATTCAATTGAAGATGGACTTCACTGCAGCAAGATTACAGAGAGGTCACTTATTACTCAAACAAGGAAAACTTGATGAAGCAGAAGATGATTTTAAAAAAGTGCTCAAATCTAATCCAAGTGAAAATGAAGAAAAGGAAGCACAGTCTCAACTTATAAAATCTGATGAAATGCAGCGTTTGCGTTCACAAGCACTTAACGCTTTTGGAAGTGGAGATTATACTGCTGCTATAGCCTTCCTTGATAAGATTTTAGAGGTTTGTGTTTGGGA

TGCAGAACTACGGGAACTTCGAGCTGAATGTTTTATAAAAGAAGGAGAACCTAGGAAAGCTATAAGTGACTTAAAAGCTGCGTCAAAGTTGAAGAATGATAATACTGAAGCGTTTTATAAAATAAGCACACTGTACTACCAACTAGGAGACCACGAACTGTCCCTCAGTGAAGTTCGGGAATGTCTTAAACTTGACCAGGATCATAAAAGGTGTTTTGCACACTATAAACAAGTAAAGAAACTTAATAAGCTGATTGAGTCAGCTGAAGAGCTCATCAGAGATGGCAGATACACAGATGCTACCAGCAAATATGAATCTGTCATGAAAACAGAGCCAAGCATTGCTGAATATACAGTTCGTTCAAAGGAGAGGATTTGCCACTGCTTTTCTAAGGACGAGAAGCCTGTTGAAGCTATTAGGGTTTGTTCTGAAGTTTTACAGATGGAACCTGACAATGTGAATGCCCTGAAAGATCGAGCAGAGGCCTATTTGATAGAGGAAATGTATGATGAAGCTATTCAGGATTATGAAACTGCTCAGGAACACAATGAAAATGATC

AGCAGATTCGAGAAGGTCTAGAGAAAGCACAAAGATTATTGAAACAGTCGCAGAAACGAGATTATTATAAAATCTTGGGAGTAAAAAGAAATGCCAAAAAGCAAGAAATTATTAAAGCATACCGAAAATTAGCACTGCAGTGGCACCCAGATAACTTCCAGAATGAAGAAGAAAAGAAAAAAGCTGAGAAAAAGTTCATTGATATAGCAGCTGCTAAAGAAGTCCTCTCTGATCCAGAAATGAGAAAGAAGTTTGACGACGGAGAAGATCCTTTGGATGCAGAGAGCCAGCAAGGAGGCGGCGGCAACCCTTTCCACAGAAGCTGGAACTCATGGCAAGGGTTCAATCCCTTCAGCTCAGGCGGACCATTTAGATTTAAATTCCACTTCAATTAG -3′

**shRNA Target Sequences：**

ShRNA 1: gcttctggccgcatgactata

ShRNA 2: gccaggtggtcaaagttaata

**Si-RNA Sequences：**

Si-RNA1: F: ggugcugaauguggaguaatt

R: uuacuccacauucagcacctt

Si-RNA2: F: ggaagcacagucucaacuutt

R: aaguugagacugugcuucctt
